# The lung response to ozone is determined by age and is partially dependent on toll-Like receptor 4

**DOI:** 10.1186/s12931-015-0279-2

**Published:** 2015-09-26

**Authors:** Kelsa Gabehart, Kelly A. Correll, Joan E. Loader, Carl W. White, Azzeddine Dakhama

**Affiliations:** Department of Pediatrics, National Jewish Health, 1400 Jackson Street, Denver, 80206 CO USA; Current address: University of Colorado Denver, Children’s Hospital, Aurora, CO USA

**Keywords:** Ozone, Age, Lung permeability, Neutrophil, Mucus, Toll-like receptor 4

## Abstract

**Background:**

Ozone pollution has adverse effects on respiratory health in children and adults. This study was carried out in the mouse model to investigate the influence of age and to define the role of toll-like receptor four (TLR4) in the lung response to ozone exposure during postnatal development.

**Methods:**

Female mice (1 to 6 weeks of age) were exposed for 3 h to ozone (1 part per million) or filtered air. Analyses were carried out at six and 24 h after completion of exposure, to assess the effects on lung permeability, airway neutrophilia, expression of antioxidants and chemokines, and mucus production. The role of TLR4 was defined by examining TLR4 expression in the lung during development, and by investigating the response to ozone in *tlr4*-deficient mice.

**Results:**

Metallothionein-1, calcitonin gene-related product, and chemokine C-X-C ligand (CXCL) five were consistent markers induced by ozone throughout development. Compared with adults, neonates expressed lower levels of pulmonary TLR4 and responded with increased mucus production, and developed an attenuated response to ozone characterized by reduced albumin leakage and neutrophil influx into the airways, and lower expression of CXCL1 and CXCL2 chemokines. Examination of the responses in *tlr4*-deficient mice indicated that ozone-mediated airway neutrophilia, but not albumin leakage or mucus production were dependent on TLR4.

**Conclusions:**

Collectively, the data demonstrate that the response to ozone is determined by age and is partially dependent on TLR4 signaling. The reduced responsiveness of the neonatal lung to ozone may be due at least in part to insufficient pulmonary TLR4 expression.

**Electronic supplementary material:**

The online version of this article (doi:10.1186/s12931-015-0279-2) contains supplementary material, which is available to authorized users.

## Introduction

Ozone (O_3_) is a common urban air pollutant generated through reaction of ultraviolet sunlight with nitrogen oxides and volatile organic compounds. O_3_ induces an influx of neutrophils into the airways and alters lung permeability leading to leakage of plasma proteins into the airways. Exposure to O_3_ reduces lung function and exacerbates asthma, resulting in increased emergency department visits and hospitalization especially in children [1–6]. Children are generally considered to be more vulnerable than adults to the adverse effects of O_3_ due to higher outdoor activity, resulting in increased exposure, and higher ventilation rates potentially leading to higher doses of inhaled O_3_ particularly in the summer. Infants are also susceptible to develop respiratory symptoms with O_3_ exposure, especially if their mothers have asthma [7]. This susceptibility to O_3_ appears to be age-related in human, at least based on the risk for asthma exacerbations [8–10]. However, because sampling of the lower airways in young children is not easily accessible, the biological response of the lung to O_3_ exposure is still not characterized for infants and children and it is not clear if this response is age-dependent in human.

The outcomes of O_3_ exposure can be influenced by many factors [11], including the concentration of O_3_, the duration and type of exposure (acute or chronic), and the conditions of exposure (rest or exercise during exposure). Both in humans and in laboratory animals, the acute pulmonary effects of O_3_, including airway neutrophilia, lung hyperpermeability and decreased lung function are normally resolved within 2 days of recovery following exposure to normal air [12–16]. Controlled exposure studies of human volunteers and laboratory animals have shown that these responses may also be attenuated after short-term, repeated daily exposure to O_3_ [17–21]. This phenomenon, referred to as tolerance or functional adaptation, is transient and may last for up to 2 weeks before full susceptibility to O_3_ with acute pulmonary effects is restored again. However, as shown in rhesus monkeys, long-term repeated cycles of O_3_ exposure can lead to morphological alterations in airway structure that are further enhanced with allergen exposure, more specifically in the early age [22].

The early age is considered to be most vulnerable to the adverse effects of air pollution because damage to the lung during this window of active growth may potentially lead to irreversible alterations in the structure and function of the airways. During the same period, particularly in the neonatal phase, the pulmonary antioxidant system undergoes maturational changes [23] and the antioxidant capacity of the lung may not be adequate enough to protect airway tissue against excessive oxidative stress generated by oxidant air pollutants such as O_3_ [24, 25].

Besides age, genetic factors linking TLR4 to the differential susceptibility to O_3_ have also been proposed [26]. TLR4 is a member of the family of toll-like receptors that recognize highly conserved molecular patterns associated with pathogens [27, 28]. TLR4 was initially characterized as the prototypic receptor for lipopolysaccharide [29], and its role in the initiation of the innate immune response is well established [30]. However, new roles have emerged for TLR4 that include the recognition of endogenous molecules called alarmins, also described as danger-associated molecular patterns, which are released upon tissue damage and trigger an inflammatory response to acute injury [31–34]. TLR4 signaling has been implicated in O_3_-mediated lung inflammation through interaction with hyaluronan fragments [35, 36], and both infection-induced and acid-induced acute lung injury also appear to be triggered by TLR4 but through interaction with oxidized phospholipids generated from oxidative stress [37].

Previous studies in mice have shown that the response to O_3_ is dose-dependent and varies with age [38, 39] In these studies, juvenile mice of 2–3 weeks of age developed a lesser degree of airway neutrophilia and lung permeability change in response to O_3_ exposure than adult (12–15 weeks old) mice. However, these responses varied also with the strain of mice used, and earlier age was not examined in these studies. Other studies examined chemokine expression in the lung of neonatal mice and found that this response was blunted even with high dose of O_3_ exposure [40]. Nonetheless, the response of the neonatal lung is still poorly characterized and it is not clear if there is a gradient of susceptibility to O_3_ from neonatal to adult age and if the responses are comparable in nature and magnitude for all developing lungs. In addition, the role of TLR4 in the various components of the response is not well defined particularly for the neonatal mice.

The objective of this study was to further investigate the age-dependent response to O_3_ exposure throughout postnatal development in mice and to define the role of TLR4 in these responses. Because pulmonary TLR4 expression appears to be developmentally regulated [41], we hypothesized that the response to O_3_ is modified by age and is partially regulated by TLR4 signaling. We examined the effect of acute O_3_ exposure on developing lungs by exposing mice of varying age from 1 to 6 weeks. We assessed the effects on lung permeability and airway neutrophilia, mucus response, and expression of chemokines, antioxidant and neuropeptides in the lung. In addition, we examined TLR4 expression and assessed the responses in both neonatal and adult TLR4^−/−^ mice.

## Materials and methods

### Animals

Wild-type (WT) BALB/c mice were obtained from the National Cancer Institute Mouse Repository (Frederick, MD). TLR4^−/−^ mice, on BALB/c background, were kindly provided by Dr. David Schwartz (University of Colorado Denver Anschutz Medical Campus, Aurora, CO). The mice were originally obtained from Dr. Shizuo Akira (Japan) [42], and were backcrossed onto BALB/c background for ten generations. The tlr4 knockout genotype was confirmed by real-time PCR using specific primers (Additional file 1: Supplemental Material). BALB/c mice were shown to be one of the most sensitive strains to O_3_ [39]. Mice were bred and maintained under specific pathogen-free conditions at the Biological Resource Center of National Jewish Health. All animals used in this study were treated humanely and in accordance with the recommendations of the National Institutes of Health Guide for the Care and Use of Laboratory Animals. All experiments were carried out under a protocol approved by the Institutional Animal Care and Use Committee. All surgeries were performed under terminal anesthesia with pentobarbital.

### Ozone exposure

O_3_ exposure was performed as previously described [43]. The O_3_ exposure system is made of two 240-liter stainless steel inhalation chambers stacked on top of one another. The upper chamber is used for O_3_ exposure and the lower is dedicated for FA exposure. All plumbing and tubing in the system that come in contact with ozone are constructed of either stainless steel or Teflon. Windows in the chambers used for animal observation during exposure are made of glass. Mice were exposed to O_3_ or FA in stainless steel mesh wire cages (Length × width × height: 25 cm × 17 cm × 17 cm) with a floor constructed of 30-mesh stainless steel mesh. This size mesh allows passage of urine, but prevents foot injury during cage loading and unloading from the chambers. Each chamber can accommodate for up to eight cages. HEPA-filtered room air was drawn into each chamber at a rate of 100 liters per min. O_3_ was generated from pure medical-grade oxygen using a silent arc discharge ozonizer (Sander Ozonizer, Model 25; Erwin Sander Elektroapparatebau GmbH, Uetze-Eltze, Germany), passed through a mass flow controller (Model 1359C; MKS Instruments Inc., Andover, MA), and introduced into the ozone exposure chamber. The concentration of O_3_ inside the chamber was continuously monitored with an ultraviolet ozone analyzer (Model 400A; Advanced Pollution Instrumentation, Inc., San Diego, CA) and recorded on a strip-chart recorder. O_3_ concentrations were stabilized by feedback control and were maintained within 5 % variation from the 1-ppm target value. Temperature inside the chambers was maintained at 20 to 25 °C. Relative humidity was maintained between 38 and 43 %. For safety, a separate ozone monitor (Model C30-Z, EcoSensors, Santa Fe, NM) was used to monitor ozone levels in the operator’s room. Should the room ozone levels exceed 100 ppb, this monitor triggers a solid-state relay that disconnects power to the ozone generator. Calibration of the ozone analyzers was performed by the Colorado Department of Public Health and Environment (Denver, CO).

### Experimental design

The following age groups of mice were studied: Neonatal (1-week old), juvenile (2-week old), weanling (3-week old), and adults (6-week old). Mice were exposed to O_3_ (1 ppm) or filtered air (FA) for 3 h. Neonatal mice (1-week old) were exposed with their mothers to minimize stress and to maintain mother/infant interaction and feeding habits. Older mice were exposed without the mothers. At the designated time points after completion of exposure, mice were euthanized by intraperitoneal injection of pentobarbital (Nembutal®, 100 mg/kg body weight) and ex-sanguinated by severing the aorta. Broncholaveolar lavage (BAL) was performed to assess airway inflammation, lung permeability change and mucus release into the airways. The lungs were processed for histology, to examine mucus expression in tissue, or for analysis of gene expression by real-time quantitative polymerase chain reaction (rt-qPCR).

### Bronchoalveolar lavage

Immediately after euthanasia, the mouse trachea was cannulated with a blunt-end needle of appropriate gauge size (23G for 1- and 2-week old, 20G for 3 and 6-week old mice). The lungs were lavaged with sterile phosphate-buffered saline (PBS), instilling 60 microliters per gram of body weight into the trachea and gently aspirating the fluid via the cannula, using 0.5-ml Hamilton glass syringe (Hamilton Inc., Reno, NV) for 1-week old neonates, or a standard 1-ml syringe (Becton Dickinson) for the other age groups. The % recovery of BAL fluid was similar for all age groups and ranged between 70 and 75 % of instilled liquid. The recovered BAL fluids were centrifuged at 485 x *g* for 5 min at 4 °C. The supernatants were collected and stored at−80 °C until needed for analyses. The cells in the pellet were suspended in 100 μl of PBS. Total cell numbers were determined by counting cells in ABC Vet hematology analyzer (Block Scientific, Bohemia, NY). For differential cell counts, an aliquot of suspended BAL cells was centrifuged onto cytospin slides for 3 min at 750 rpm using Shandon Cytospin3 centrifuge (Fisher Scientific, Hanover Park, IL). The cytospin preparations were air-dried and stained with Leukostat (Fisher Diagnostics, Pittsburgh, PA) to differentiate inflammatory cells by standard hematological procedures. Neutrophil counts were determined as percent of total BAL cells and were expressed as absolute numbers of recovered cells.

### Measurement of albumin and Muc5ac levels in the BAL fluid

The levels of albumin (Bethyl Laboratories, Montgomery, TX) and Muc-5 ac (TSZ ELISA, Framingham, MA) were measured in the BAL fluid, using commercial ELISA. The detection limits were 7.8 ng/ml for albumin, and 4.7 ng/ml for Muc5ac.

### Detection and quantification of mucus in tissue

Immediately after lavage, the lungs were inflated with paraformaldehyde (4 % in PBS), administered through the tracheal cannula at 20-cm of static fluid pressure, removed and immersed in the same fixative for 24 h at 4 °C. Tissues were dehydrated in graded ethanols, cleared in xylene and embedded in paraffin. Mucin-5 AC (Muc5ac) was detected by immunohistochemistry using a specific mouse monoclonal antibody (Clone 45 M1) (Thermo Scientific, Fremont, CA) and the ImmPRESS anti-mouse Immunoglobulin (peroxidase) Polymer detection kit (Vector Laboratories, Burlingame, CA). Muc5ac expression in central intrapulmonary airways was quantified by morphometry using ImageJ analysis program (version 1.34 s for Macintosh) developed at the US National Institutes of Health and available via the Internet at the public domain http://rsb.info.nih.gov. Briefly, images of stained tissue sections were captured with Olympus DP72 digital camera under Olympus BX51 microscope (Leeds Precision Instruments, Minneapolis, MN). The images were transferred to the computer and calibrated against a micrometric scale to convert digital pixels to numeric distance. With ImageJ program, the area of Muc5ac staining was outlined automatically and measured after adjusting the threshold function to highlight the pixels matching Muc5ac staining within the epithelium. In parallel, the adjacent basement membrane (BM) of the epithelium was outlined manually using the freehand line selection tool and was measured. The results are expressed as Muc5ac immunoreactive area normalized to the length of the corresponding epithelium basement membrane (μm^2^/mm BM).

### Analysis of gene expression in lung tissue

RNA expression analysis was performed on lung tissue. Freshly isolated lungs were stabilized in RNA*later* (Qiagen, Valencia, CA). Total RNA was isolated using TRIzol® reagent (Life Technologies, Grand Island, NY) and was cleaned up using RNeasy Mini Kit (Qiagen). Total RNA (1 μg) was transcribed into cDNA using M-MLV Reverse Transcriptase and oligo-(dT)_12–18_ primers (Life Technologies) for 30 min at 42 °C in a 20-μl reaction volume. One microliter of transcribed cDNA was amplified by real-time qPCR using Taqman Universal PCR Master Mix. The expression of CXCL1, CXCL2, CXCL5, HMOX1, GSR, MT1, NRF2, TLR4, and GAPDH was analyzed by qPCR using Taqman Gene Expression assays (Life Technologies). The expression of the sensory neuropeptides CGRP and substance P was analyzed using custom designed primers and probes (Table 1). Real-time qPCR was performed in a 25-μl reaction volume using ABI Prism® 7000 Sequence Detection System (Applied Biosystems, Foster City, CA). Temperature cycling conditions included 1 cycle of heating to 95 °C for 10 min, followed by 40 cycles of denaturation at 95 °C for 10 s and annealing/extension at 60 °C for 1 min. Data are presented as target gene expression level relative to GAPDH housekeeping gene expression (2^-deltaCt^), where delta Ct represents the difference between the Ct (cycle threshold) value of target gene and the Ct value of GAPDH. The Ct values of GAPDH were similar for all groups; hence GAPDH was used for normalization of qPCR data.

**Table 1:** Primers and probes designed for detection of CGRP and substance P

CGRP	
Accession number	AF330212
Forward primer (position 302)	5’-AGGAGGCTGAGGGCTCTAGTG-3’
Reverse primer (position 358)	5’- CAGCCGATGGGTCACACA-3’
Internal probe (FAM-labeled)	5’-CTGCTCAGAAGAGATCCTGCAACACTGC-3’
Substance P	
Accession number	NM_009311
Forward primer (position 212)	5’-GACCAGATCAAGGAGGCAATG-3’
Reverse primer (position 260)	5’-GGGTCTTCGGGCGATTCT-3’
Internal probe (TET-labeled)	5’-CGGAGCCCTTTGAGCATCTTCTGCA-3’

### Statistical analysis

Data are presented as mean ± SEM. Statistical analysis was performed using GraphPad Prism version 5.0 for Mac OS X (GraphPad Software, San Diego, CA). Data were analyzed using ANOVA with Bonferroni’s procedure to correct for multiple comparisons between the groups. Student’s *t*-test was used for all comparisons between group pairs. Statistical significance was defined as a *p* value of < 0.05.

## Results

### Expression of antioxidants and sensory neuropeptides

**Fig. 1:**
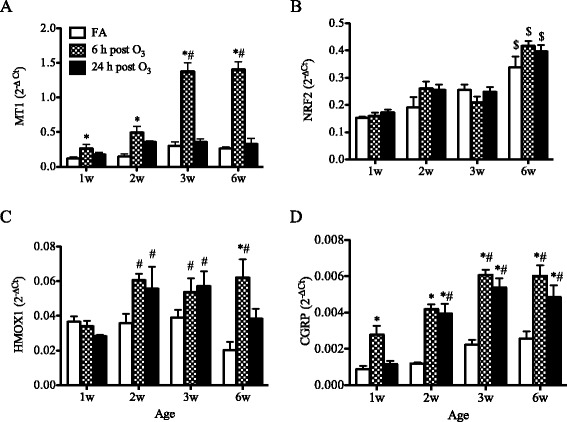
Age-related effects of O_3_ on antioxidant and neuropeptide gene expression in the lung. BALB/c mice of varying age, from 1 to 6 weeks, were exposed to FA or O_3_ for 3 h. Expression of MT1 (**a**), NRF2 (**b**), HMOX1 (**c**) and CGRP (**d**) was analyzed in the lungs by real-time qPCR. Data are normalized to GAPDH and presented as mean ± SEM (*n* = 3-6 mice/group). *: *p* < 0.05, compared with age-matched FA-exposed controls; #: *p* < 0.05, compared with 1-week old group; $: *p* < 0.05, compared to all other age groups. MT1: metallothionein-1, NRF2: Nuclear factor (erythroid-derived 2)-like2, HMOX1: Heme oxygenase-1, CGRP: Calcitonin gene-related peptide

O_3_ induced significant increases in MT1 expression at 6 h post exposure in the lungs of all age groups examined (Fig. 1a). By 24 h, MT1 expression returned to baseline levels similar to those detected in FA-exposed animals. The results also show that O_3_-mediated MT1 expression correlated with age and was significantly higher in 3- and 6-week old mice compared to 1- and 2-week old mice. Regardless of exposure, NRF2 expression increased gradually with age and reached significantly higher levels in the 6-week old mice compared to all other age groups (Fig. 1b). However, this baseline expression was not altered by O_3_ exposure in any age group (Fig. 1b). The expression of HMOX1 (Fig. 1c) and GSR (Additional file 2: Figure S1), two antioxidant genes regulated by NRF2, did not increase after O3 exposure in the neonatal lungs. In older mice, compared to neonatal mice, both antioxidant responses were significantly higher at 24 h post-O_3_ exposure, while the induced levels were similar in 2, 3 and 6 weeks old mice (Fig. 1c, Additional file 2: Figure S1).

Previous observations from this laboratory indicated that O_3_ increases CGRP expression but does not alter substance P expression in the neonatal mouse lung [44]. In the present study, pulmonary substance P expression was not altered after O_3_ exposure in any age group examined (data not shown). In contrast, the expression of CGRP was significantly increased in all age groups at 6 h following O_3_ exposure, compared to FA exposure (Fig. 1d). This increase in pulmonary CGRP expression was transient in the 1-week old mouse lung, whereas in the juvenile and adult mouse lungs it increased at 6 h and was maintained up to 24 h after O_3_ exposure.

### Lung permeability and airway neutrophilia

**Fig. 2:**
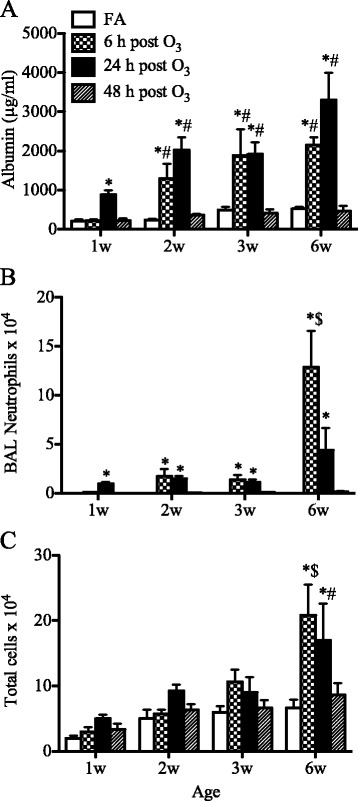
Age-related effects of O_3_ on lung permeability and airway neutrophilia. Levels of albumin (**a**), neutrophil counts (**b**), and total cell numbers (**c**) were determined in the BAL fluids after exposure to O_3_ or FA. Values are mean ± SEM (*n* = 3–6 mice/group). *: *p* < 0.05, compared with age-matched FA-exposed controls; #: *p* < 0.05, compared with 1-week old group; $: *p* < 0.05, compared to all other age groups

Altered lung permeability and airway neutrophilia are characteristic of O_3_-mediated lung injury. To establish the role of age in the development of these alterations, we examined the responses in the different age groups of mice. O_3_ mediated significant increases in albumin levels in the airways of mice from 1 to 6 weeks of age (Fig. 2a). In juvenile and adult mice, the albumin levels increased significantly at 6 h and were maintained at 24 h after O_3_ exposure. By contrast, in the 1-week old neonates, this albumin leakage was less prominent than in older mice and appeared to be delayed to 24 h after O_3_ exposure. At 48 h post-O_3_ exposure, the levels of albumin were similar to baseline levels for all age groups.

As shown in Fig. 2b, O_3_ induced a significant influx of neutrophils into the airways of mice in all age groups. Interestingly, the extent and pattern of this response varied considerably with age. In the 1-week old neonatal mice, the neutrophilic response was markedly attenuated compared to the response that developed in the adult lung. In 2- and 3-week old mice, the numbers of neutrophils were significantly elevated at 6 h and were maintained with no apparent decline at 24 h post-O_3_ exposure. By contrast, in adults, the neutrophil numbers were much higher at 6 h but declined rapidly by 24 h after O_3_ exposure. By 48 h post-O_3_ exposure, the numbers of neutrophils were similar to those recovered in the BAL fluids of FA-exposed animals in all age groups.

Figure 2c illustrates the total number of cells recovered in the BAL fluids from all age groups. In mice aged 1 to 3 weeks, O_3_ exposure produced only a small increase in total BAL cells that did not reach statistical significance when compared to the total numbers recovered from FA-exposed animals. By contract, in 6-week old mice, O_3_ mediated a significant increase in total cells that reached about 2-fold the total numbers recovered from FA-exposed animals. By 48 h post-exposure, the total cell numbers recovered from O_3_-exposed animals were similar to the total numbers of cells recovered from FA-exposed animals in all age groups.

### Expression of neutrophilic chemokines

**Fig. 3:**
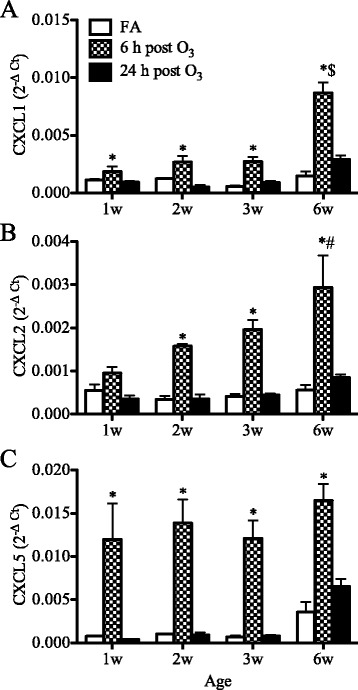
Age-related effects of O_3_ on neutrophilic chemokine expression in the lung. Expression of CXCL1 (**a**), CXCL2 (**b**) and CXCL5 (**c**) was analyzed in the lungs by real-time qPCR at 6 and 24 h after completion of exposure. Data are normalized to GAPDH and presented as mean ± SEM (*n* = 3–6 mice/group). *: *p* < 0.05, compared with age-matched FA-exposed controls; #: *p* < 0.05, compared with 1-week old group; $: *p* < 0.05, compared to all other age groups

To further investigate the age-dependent neutrophilic response to O_3_, we examined the expression of the neutrophilic chemokines CXCL1, CXCL2 and CXCL5 in the lungs by real-time qPCR. In all age groups, O_3_ induced a transient increase in the expression of all three chemokines notably at 6 h post-exposure (Fig. 3). This increase appeared to be age-dependent for CXCL1 and CXCL2, with significantly higher expression induced in the adult lung compared to the neonatal lung (Fig. 3a-b). By contrast, independent of age, O_3_ induced significant increases in CXCL5 expression to similar levels all age groups from neonates to adults (Fig. 3c).

### Airway mucus production

**Fig. 4:**
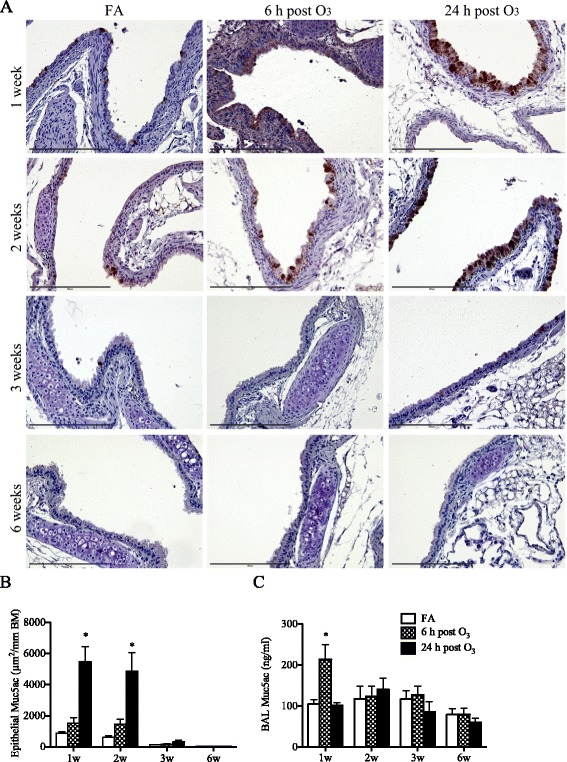
Age-related effects of O_3_ on airway mucus expression and release. (**a**) Muc5ac was detected in tissue of central intrapulmonary airways by immunohistochemistry (brown staining) and was localized to the airway epithelium. Scale bar represents 200 μm. (**b**) Epithelial Muc5ac expression was quantified by morphometry. The data are expressed as area of Muc5ac-positive epithelium normalized to the perimeter of the epithelial basement membrane (mean ± SEM, *n* = 3–4/group). (**c**) Levels of Muc5ac measured in the BAL fluids (mean ± SEM, *n* = 3-7/group). *: *p* < 0.05, compared with age-matched FA-exposed controls

Mucus expression was examined in tissue of central intrapulmonary airways by immunostaining for Muc5ac and was quantified by morphometry. In parallel, mucus release was estimated by measurement of Muc5ac levels in the recovered BAL fluids. As illustrated in Fig. 4a, Muc5ac was constitutively expressed in the airways of FA-exposed neonatal mice, and this constitutive expression declined progressively as the lungs developed with age (Fig. 4a, b). Following O_3_ exposure, the expression of Muc5ac further increased significantly in the airways of 1- and 2-week old mice, most notably at 24 h after exposure (Fig. 4a, b). However, as shown for older mice, this increase was progressively attenuated with age. In the BAL fluid, the levels of Muc5ac were significantly increased at 6 h post-O_3_ exposure in neonatal mice but not in juvenile or adult mice (Fig. 4c). By 24 h post-O_3_ exposure, the levels returned to baseline values similar to those measured in FA-exposed controls.

### Role of TLR4 in the age-dependent response to O_3_

The reason why neonatal mice appear to be less responsive to O_3_ than adults is not clear. However, neonatal mice express lower levels of TLR4 in the lung compared to adult mice [41], and TLR4 appears to be required for the inflammatory response to O_3_ that develops in the adult mouse lung [45]. Therefore, we reasoned that the attenuated response to O_3_ observed in the neonatal lung might be due at least in part to a reduced expression of pulmonary TLR4 in this early age.

**Fig. 5:**
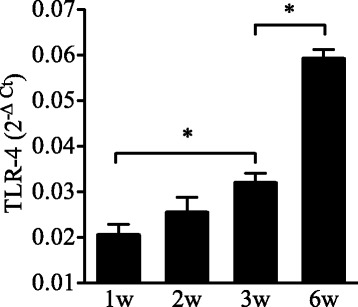
Age-related change in pulmonary TLR4 expression. TLR4 expression was determined by real-time qPCR in whole lung tissue. Data are normalized to GAPDH and are presented as mean ± SEM (*n* = 4–14 mice/group). *: Statistical difference between the age groups (*p* < 0.05)

To determine if the age-dependent response to O_3_ is related to differences in pulmonary TLR4 expression, we first examined the expression of TLR4 in the lungs of developing mice from neonates to adults. As shown in Fig. 5, consistent with previous observations [41], the expression of pulmonary TLR4 increased in an age-related manner from 1 week to 6 weeks of postnatal development, with the lowest expression levels detected in the neonatal lung and highest levels in the adult lung.

To determine which component of the response to O_3_ is dependent on TLR4 and if TLR4 deficiency recapitulates some aspects of the response seen in the neonates, we investigated the response to O_3_ in both neonatal and adult TLR4^−/−^ mice and compared the results to those obtained in WT mice of similar age.

**Fig. 6:**
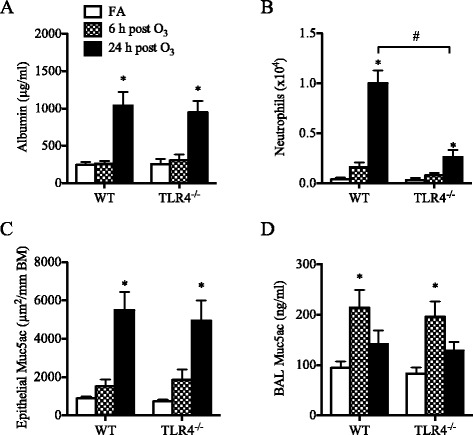
Effect of O_3_ on lung permeability, airway neutrophilia, and mucus production in neonatal (1-week old) TLR4^−/−^ mice compared to wild-type (WT) mice. (**a**) Levels of albumin measured in the BAL fluid (mean ± SEM, *n* = 3–5/group), (**b**) Numbers of neutrophils recovered in the BAL fluids (mean ± SEM, *n* = 3–5/group), (**c**) Epithelial Muc5ac expression (mean ± SEM, *n* = 3–5/group), (**d**) Levels of Muc5ac in the BAL fluid (mean ± SEM, *n* = 3–5/group). *: *p* < 0.05, compared with age-matched FA-exposed controls; #: Statistical difference between the groups (*p* < 0.05)

**Fig. 7:**
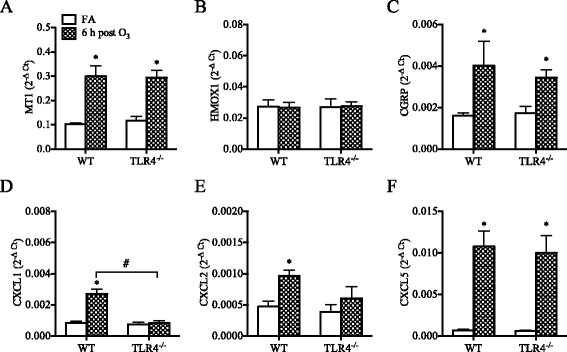
Effect of O_3_ on pulmonary antioxidant, neuropeptide, and chemokines expression in neonatal (1-week old) TLR4^−/−^ mice compared to wild-type (WT) mice. (**a**) metallothionein-1 (MT1), (**b**) heme oxygenase-1 (HMOX-1), (**c**) calcitonin gene-related peptide (CGRP), (**d**) chemokine (C-X-C) ligand 1 (CXCL1), (**e**) chemokine (C-X-C) ligand 2 (CXCL2), (**f**) chemokine (C-X-C) ligand 5 (CXCL5). Expression was analyzed by real-time qPCR. Data are normalized to GAPDH and presented as mean ± SEM (*n* = 3–5 mice/group). *: *p* < 0.05, compared with age-matched FA-exposed controls; #: Statistical difference between the groups (*p* < 0.05)

When neonatal (1-week old) TLR4^−/−^ mice were exposed to O_3_ they developed significant increases in lung permeability similar to O_3_-exposed neonatal WT mice (Fig. 6a), but their neutrophilic response was markedly attenuated at 24 h (Fig. 6b) and subsided by 48 h post-O_3_ exposure (Additional file 3: Figure S2A). Other responses to O_3_, including mucus production (Fig. 6c, d), antioxidant response (Fig. 7a, b, Additional file 4: Figure S3A), and CGRP expression (Fig. 7c) developed independent of TLR4 expression in the neonatal lung. Interestingly, in line with the attenuated neutrophilic response, no significant increase in CXCL1 or CXCL2 expression was observed after O_3_ exposure in the lungs of neonatal TLR4^−/−^ mice (Fig. 7d, e), although the expression of CXCL5 was significantly increased in the lungs of these mice (Fig. 7f).

**Fig. 8:**
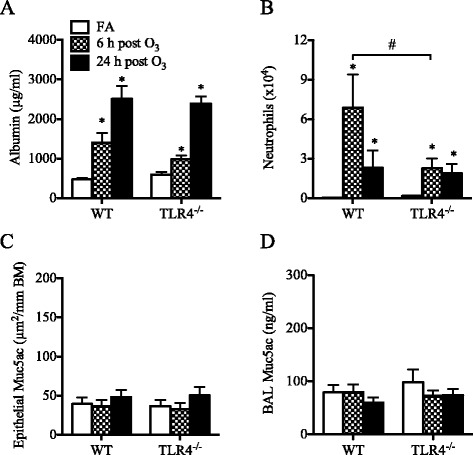
Effect of O_3_ on lung permeability, airway neutrophilia, and mucus production in adult (6-week old) TLR4^−/−^ mice compared to wild-type (WT) mice. (**a**) Albumin levels measured in the BAL fluids (mean ± SEM, *n* = 4–7/group), (**b**) Number of neutrophils recovered in the BAL fluids (mean ± SEM, *n* = 4–7/group), (**c**) Epithelial Muc5ac expression (mean ± SEM, *n* = 3–6/group), (**d**) Muc5ac levels in the BAL fluids (mean ± SEM, *n* = 5–10/group). *: *p* < 0.05, compared with age-matched FA-exposed controls; #: Statistical difference between the groups (*p* < 0.05)

**Fig. 9:**
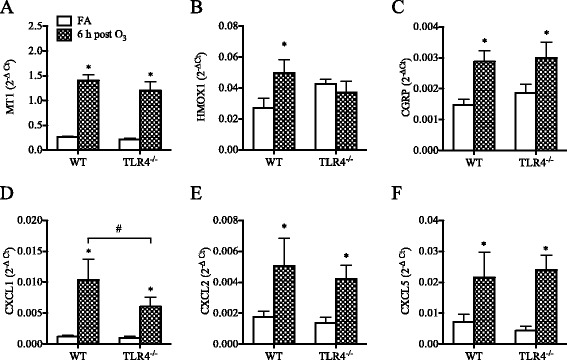
Effect of O_3_ on pulmonary antioxidant, neuropeptide, and neutrophilic chemokines expression in adult (6-week old) TLR4^−/−^ mice compared to wild-type (WT) mice. (**a**) metallothionein-1 (MT1), (**b**) heme oxygenase-1 (HMOX-1), (**c**) calcitonin gene-related peptide (CGRP), (**d**) chemokine (C-X-C) ligand 1 (CXCL1), (**e**) chemokine (C-X-C) ligand 2 (CXCL2), (**f**) chemokine (C-X-C) ligand 5 (CXCL5). Expression was analyzed by real-time qPCR and was normalized to GAPDH. Data are presented as mean ± SEM (*n* = 5–6 mice/group). *: *p* < 0.05, compared with age-matched FA-exposed controls; #: Statistical difference between the groups (*p* < 0.05)

Similarly, in 6-week old adult mice, TLR4-deficiency did not alter the effect of O_3_ on lung permeability (Fig. 8a) but airway neutrophilia was significantly reduced in TLR4^−/−^ mice compared to WT mice (Fig. 8b, Additional file 3: Figure S2B). Examination of airway tissue showed no increase in Muc5ac expression in the airways (Fig. 8b), and Muc5ac levels were not increased after O_3_ exposure in the BAL fluid of these mice (Fig. 8c), a response that was of a low magnitude similar to the response of adult WT mice. Analysis of antioxidant response showed no difference between adult TLR4^−/−^ and adult WT mice in O_3_-mediated MT1 expression (Fig. 9a). However, unlike in adult WT mice, neither HMOX1 nor GSR was increased after O_3_ exposure in the lungs of adult TLR4^−/−^ mice (Fig. 9b, Additional file 4: Figure S3B). TLR4-deficiency did not affect the O_3_-mediated pulmonary expression of CGRP (Fig. 9c). Finally, the expression of CXCL1 but not CXCL2 or CXCL5 was significantly reduced after O_3_ exposure in the lungs of adult TLR4−/− mice compared with adult WT mice (Fig. 9d–f).

## Discussion

The results of this study demonstrate that the lung response to acute O_3_ exposure is determined by age and is partially dependent on TLR4 signaling. In neonatal mice, compared to adult mice, this response was markedly attenuated, with lower expression of antioxidants, reduced albumin leakage, and reduced neutrophils influx into the airways associated with lower expression of the neutrophilic chemokines CXCL1 and CXCL2. In contrast, mucus production was higher at baseline and increased significantly following acute O_3_ exposure in the neonatal lung but not in the adult lung. Interestingly, the expression of pulmonary TLR4 appears to be developmentally regulated, with the neonatal lung expressing much lower levels of TLR4 compared to the adult lung, and examination of the response in neonatal TLR4^−/−^ mice revealed that TLR4 signaling is required for the development of airway neutrophilia but not albumin leakage or mucus production in response to acute O_3_ exposure. In adult mice, TLR4 is also implicated in the neutrophilic airway response to O_3_ but not in albumin leakage. These results may explain why the neonatal lung is less responsive to O_3_ exposure compared to fully developed, adult lung. Whether the response to O_3_ is similarly age-dependent in the developing human lung remains to be determined. However, due to many ethical limitations, the nature and magnitude of the biological response that might develop in the newborn human lung after O_3_ exposure may not be answered directly.

Experimental exposure studies have shown that the biologic pulmonary response to O_3_ is closely linked to the alveolar dose of inhaled O_3_ in both human and rodents, which is related to the subject’s alveolar ventilation and is proportional to the amount of O_3_ breathed [46–50]. This linkage appears to be preserved across species allowing for extrapolation of O_3_ dose and effect from rodents to humans [49, 50]. Initial studies in rats reported excessively higher mortality rates in neonates (1-week old) compared to older animals after 3 days of continuous exposure to 0.8 ppm O_3_ [51, 52]. However, in the present study, mortality was not observed in any age group of mice, including neonates, after short-term (3 h) exposure to a similar dose of 1 ppm O_3_.

Neutrophils are common biological response markers of O_3_ exposure in human and rodents. Neutrophils are recruited to the lung through a coordinated action of chemokines. When examined in this study, CXCL1 and CXCL2 were barely induced by O_3_ in the first week of life but their expression increased significantly at later ages. By contrast, the expression of CXCL5 was markedly increased to similar levels in all age groups including neonates, and there was no correlation with the age-related neutrophilic response to O_3_. A recent study uncovered an unexpected and important role for CXCL5 as a negative regulator of neutrophil trafficking in the lung [53]. The role of CXCL5 in our model is not clear, but according to this new paradigm it may also play a regulatory role by limiting the extent of neutrophil recruitment to the lung during O_3_ exposure.

The production of mucus in response to O_3_ exposure has not, to our knowledge, been studied in the context of the developing lung. The increased levels of Muc5ac detected after O_3_ exposure in the BAL fluid of neonatal mice, but not in older mice, indicate that mucus release is a unique feature of the neonatal lung response to O_3_. This response may be partially explained by the high constitutive number of goblet cells present in the airways of neonatal mice compared with adults, as shown in this study and by others [54]. As result of this constitutive mucus production, the composition of the epithelial lining fluid is likely to be different in the neonatal lung compared to the adult lung. When inhaled into the airways, O_3_ interacts with proteins and lipids present in the epithelial lining fluid or on the surface of epithelial cells and mediates its toxic effects partly through the generation of reactive oxygen products that cause tissue injury [55]. Using labeled ozone (^18^O_3_) to quantify reaction products of O_3_ with lung tissue, Vancza et al. [39] demonstrated that the amount of O_3_ absorbed in lung tissue following exposure is lower in neonatal mice compared to adult mice. Accordingly, the lower response of neonatal mice to O_3_ could be due in part to a lower O_3_ effective dose received in their lungs. On the other hand, one of the functions of the mucus layers lining the respiratory tract is thought to be the scavenging of highly reactive oxygen species, which would provide antioxidant protection to the underlying epithelium [56, 57]. Accordingly, mucus could play a cytoprotective role against oxidant air pollutants such as O_3_, which remains to be fully investigated in future studies.

The susceptibility to O_3_ is thought to be genetically determined given the marked individual variability in the reported effects of O_3_ on respiratory health in human and in different strains of mice [58, 59]. This notion is supported by genetic studies in mice [60, 61] and the subsequent identification of *tlr4* as a candidate gene for the susceptibility to O_3_ [45]. Examination of pulmonary TLR4 expression in the various mouse age groups revealed that TLR4 is developmentally regulated and is expressed at significantly lower levels in the neonatal lung compared to the adult lung. This led us to further investigate the effects of O_3_ exposure in both neonatal and adult TLR4^−/−^ mice to determine which component of the response is dependent on TLR4 and if TLR4 deficiency recapitulates some aspects of the attenuated neonatal lung response to O_3_. Among the responses examined, it was mainly the neutrophilic response and associated chemokines (CXCL1) that were dependent on TLR4 during O_3_ exposure in both age groups. The observation that HMOX1 and GSR were not increased after O_3_ exposure in adult TLR4^−/−^ mice suggests that TLR4 signaling may be involved in the induction of these responses by sensing reactive products of oxidative stress during O_3_ exposure. TLR4 signaling has shown to be required for HMOX-1 expression in LPS-induced liver injury [62] and in hemorrhage-induced lung injury [63]. This dependence on TLR4, at least for HMOX-1, could explain why HMOX-1 expression was not upregulated after O_3_ exposure in the neonatal lung where TLR4 expression is insufficient. However, other studies showed that newborn mice do not upregulate HMOX-1 expression in their lungs, but adults do, following exposure to hyperoxia [64, 65], apparently because newborn mice express high levels of Bach1 [66], a negative regulator of HMOX-1 gene transcription [67]. Our findings may suggest that, although Bach1 may repress HMOX-1 gene expression, there is also insufficient TLR4 signaling to drive HMOX-1 expression in the neonatal lung. Other TLR4-idependent responses included the potential markers of O_3_ exposure, i.e. MT1, CGRP and CXCL5. Importantly, TLR4 was not required for O_3_-mediated mucus production in the neonatal lung, and it was dispensable for O_3_-mediated lung permeability, as previously shown by other studies in adult mice [35, 36, 68, 69].

## Conclusions

The results of this study demonstrate that the response of the lung to acute O_3_ exposure is determined by age and is partially regulated by TLR4 signaling. In particular, the neonatal lung responds to O_3_ exposure differently than the adult lung, with increased mucus production and markedly attenuated airway neutrophilia and antioxidant response. Neonates, compared to adults, express lower levels of pulmonary TLR4, which may explain their reduced airway neutrophilia and antioxidant response to acute O_3_ exposure. This study also identified biological response markers potentially useful for assessing O_3_ exposure throughout postnatal lung development. These results may be important to understanding the effects of air pollution and the response of the developing lung in the early age.

## Additional files

Additional file 1:
**Supplemental Material.** (DOCX 49 kb) Additional file 2: Figure S1. Age-related effect of O_3_ on Glutathione reductase (GSR) expression. GSR expression was analyzed in lung tissue of mice (1 to 6 weeks of age) by real-time qPCR. Data are normalized to GAPDH and presented as mean ± SEM (*n* = 3–5 mice/group). *: *p* < 0.05, compared with age-matched FA-exposed controls; #: *p* < 0.05, compared with 1-week old group. (EPS 115 kb) Additional file 3: Figure S2. BAL neutrophil counts at 48 h post-O_3_exposure in WT and TLR4^−/−^ mice. WT and TLR4^−/−^ mice were exposed as neonates (A) or adults (B), to O_3_ or filtered air (FA). In both age groups, O_3_-mediated neutrophil response was not delayed in TLR4^−/−^ mice compared to WT mice. NS: No significant difference, compared to FA exposed group (*n* = 4–6 mice/group). (EPS 113 kb) Additional file 4: Figure S3. Effect of O_3_ on GSR expression in the lungs of WT and TLR4^−/−^ mice. WT and TLR4^−/−^ mice were exposed as neonates (A) or adults (B), to O_3_ or filtered air (FA). GSR expression was analyzed by rt-qPCR at 6 h post-exposure. Data are mean ± SEM values of GSR expression, normalized to GAPDH expression (*n* = 3–5 mice/group). *: *p* < 0.05, compared with FA-exposed control group. (EPS 101 kb)

## Abbreviations

BAL: Bronchoalveolar lavage
CGRP: Calcitonin gene-related peptide
CXCL1: Chemokine (C-X-C) ligand 1
CXCL2: Chemokine (C-X-C) ligand 2
CXCL5: Chemokine (C-X-C) ligand 5
FA: Filtered air
GAPDH: Glyceraldehyde-3-phosphate dehydrogenase
GSR: Glutathione reductase
HMOX1: Heme oxygenase 1
Muc5ac: Mucin 5 AC
MT1: Metallothionein 1
NRF2: Nuclear factor (erythroid-derived 2)-like 2
O_3_: Ozone
PAS: Periodic acid-schiff
PBS: Phosphate buffered saline
ppm: Part per million
rt-qPCR: real-time quantitative polymerase chain reaction
TLR4: Toll-like receptor 4
TLR4^−/−^: TLR4 knock-out
WT: Wild-type
