# Supplementary material for: The lung response to ozone is determined by age and is partially dependent on toll-Like receptor 4
Source: Respir Res. 2015 Sep 26;16:117. doi: 10.1186/s12931-015-0279-2 (PMC4583721; doi:10.1186/s12931-015-0279-2)
Supplement: Additional file 1: — Supplemental Material. (DOCX 49 kb) [file 12931_2015_279_MOESM1_ESM.docx]

**Supplemental Material**

**The lung response to ozone is determined by age and is partially dependent on Toll-Like Receptor 4.**

Kelsa Gabehart^1^, Kelly A. Correll^1^, Joan E. Loader^1¥^, Carl W. White^1¥^, Azzeddine Dakhama^1*^

^1^Department of Pediatrics, National Jewish Health, Denver, Colorado, U.S.A.

^¥^Current address: University of Colorado Denver, Children’s Hospital, Aurora, Colorado, U.S.A.

**MATERIALS AND METHODS**

***Mouse genotyping****.* TLR4^-/-^ mice were genotyped by multiplex PCR using the following oligonucleotide primers: 5’ CTG ACG AAC CTA GTA CAT GTG GA 3’ (TLR4 Forward), 5’ ACC TCT TAG AGT CAG TTC ATG GA 3’ (TLR4 Reverse), 5’ ATG GCC TTC TTG ACG AGT TC 3’ (TLR4 neomycin). DNA was extracted from mouse ear punch or tail snips and amplified using the REDExtract-N-Amp^TM^ Tissue PCR kit, as instructed by the manufacturer (Sigma-Aldrich, St-Louis, MO). The PCR products were resolved by electrophoresis on 2% agarose gel yielding specific bands of 160 base pairs for TLR4^-/-^ mice and 185 base pairs for wild-type mice.

**FIGURE LEGENDS**

**Supplement Figure 1**. Age-related effect of O_3_ on Glutathione reductase (GSR) expression. GSR expression was analyzed in lung tissue of mice (1 to 6 weeks of age) by real-time qPCR. Data are normalized to GAPDH and presented as mean ± SEM (n=3-5 mice/group). *: p < 0.05, compared with age-matched FA-exposed controls; #: p < 0.05, compared with 1-week old group.

**Supplement Figure 2**. BAL neutrophil counts at 48 h post-O_3_ exposure in WT and TLR4^-/-^ mice. WT and TLR4^-/-^ mice were exposed as neonates (**A**) or adults (**B**), to O_3_ or filtered air (FA). In both age groups, O_3_-mediated neutrophil response was not delayed in TLR4^-/-^ mice compared to WT mice. NS: No significant difference, compared to FA exposed group (n=4-6 mice/group).

**Supplement Figure 3**. Effect of O_3_ on GSR expression in the lungs of WT and TLR4^-/-^ mice. WT and TLR4^-/-^ mice were exposed as neonates (**A**) or adults (**B**), to O_3_ or filtered air (FA). GSR expression was analyzed by rt-qPCR at 6 h post-exposure. Data are mean ± SEM values of GSR expression, normalized to GAPDH expression (n=3-5 mice/group). *: p < 0.05, compared with FA-exposed control group.
